# Sensory Neurons Arouse *C*. *elegans* Locomotion via Both Glutamate and Neuropeptide Release

**DOI:** 10.1371/journal.pgen.1005359

**Published:** 2015-07-08

**Authors:** Seungwon Choi, Kelsey P. Taylor, Marios Chatzigeorgiou, Zhitao Hu, William R. Schafer, Joshua M. Kaplan

**Affiliations:** 1 Department of Molecular Biology, Massachusetts General Hospital, Boston, Massachusetts, United States of America; 2 Department of Neurobiology, Harvard Medical School, Boston, Massachusetts, United States of America; 3 Biological and Biomedical Sciences program, Harvard Medical School, Boston, Massachusetts, United States of America; 4 Cell Biology Division, MRC Laboratory of Molecular Biology, Cambridge, United Kingdom; University of California San Diego, UNITED STATES

## Abstract

*C*. *elegans* undergoes periods of behavioral quiescence during larval molts (termed lethargus) and as adults. Little is known about the circuit mechanisms that establish these quiescent states. Lethargus and adult locomotion quiescence is dramatically reduced in mutants lacking the neuropeptide receptor NPR-1. Here, we show that the aroused locomotion of *npr-1* mutants results from the exaggerated activity in multiple classes of sensory neurons, including nociceptive (ASH), touch sensitive (ALM and PLM), and stretch sensing (DVA) neurons. These sensory neurons accelerate locomotion via both neuropeptide and glutamate release. The relative contribution of these sensory neurons to arousal differs between larval molts and adults. Our results suggest that a broad network of sensory neurons dictates transitions between aroused and quiescent behavioral states.

## Author Summary

Animals switch between periods of behavioral arousal and quiescence in response to environmental, developmental, and circadian cues. Little is known about the circuit mechanisms that produce these behavioral states. During larval molts, *C*. *elegans* exhibits a sleep-like state (termed lethargus) that is characterized by the absence of feeding and profound locomotion quiescence. We previously showed that mutants lacking the neuropeptide receptor NPR-1 exhibit increased arousal during larval molts, which is in part mediated by increased secretion of an arousal peptide (PDF-1). Here, we compare the circuits regulating arousal in larval molts and adults. We show that a broad network of sensory neurons arouses locomotion but that the impact of each neuron differs between lethargus and adults. We propose that this broad sensory network allows *C*. *elegans* to adapt its behavior across a broad range of developmental and physiological circumstances.

## Introduction

Animals undergo periods of behavioral quiescence and arousal in response to changes in their environment and metabolic state. Arousal is defined as a state of heightened responsiveness to external stimuli coupled with increased motor activity whereas quiescence is associated with diminished responsiveness and motor activity [1]. Quiescence and arousal can persist for minutes to hours. Arousal is associated with fear, stress, hunger, and exposure to sexual partners [1], while quiescence is associated with sleep and satiety [2]. Relatively little is known about the specific circuit mechanisms leading to arousal or quiescence. In particular, it is unclear if similar mechanisms mediate quiescence and arousal in response to different cues, or at different times during development. To address this question, we have analyzed arousal and quiescence of *C*. *elegans* locomotion.

During each larval molt, *C*.*elegans* undergoes a prolonged period of profound behavioral quiescence, termed lethargus behavior, whereby locomotion and feeding behaviors are inactive for approximately 2 hours [3]. Lethargus has properties of a sleep-like state such as reduced sensory responsiveness and homeostatic rebound of quiescence following perturbation [4]. Several genes and molecular pathways involved in lethargus behavior have been identified [4–11]. Multiple sensory responses are diminished during lethargus, including those mediated by a nociceptive neuron (ASH) [12], and by mechanosensory neurons [11,13].

Mutants lacking NPR-1 Neuropeptide Y (NPY) receptors have been utilized as a model for generalized arousal. NPR-1 inhibits the activity of a central sensory circuit that is defined by gap junctions to the RMG interneuron [14]. In *npr-1* mutants, responses mediated by the RMG circuit (e.g. pheromone and oxygen avoidance) are exaggerated, and this heightened acuity is associated with exaggerated locomotion (both during lethargus and in adults) [11,14–16]. Mutations that increase (e.g. *npr-1*) and decrease (e.g. *tax-4* CNG and *osm-9* TRPV) RMG circuit activity are associated with locomotion arousal and quiescence respectively [11,14,17,18].

We previously showed that locomotion quiescence during lethargus is dramatically reduced in *npr-1* mutants and that this effect requires increased RMG sensory activity [11]. Subsequent studies showed that in microfluidic chambers *npr-1* mutants have modest defects in lethargus quiescence when sensory cues are minimized but that dramatic quiescence defects are observed following brief stimulation with light or vibration [19,20]. Taken together, these papers suggest that *npr-1* mutants exhibit aroused locomotion as a consequence of enhanced sensory activity.

The arousing effects of the RMG circuit are mediated in part by secretion of a neuropeptide, pigment dispersing factor (PDF-1) [11]. Activation of PDF receptors (PDFR-1) in peripheral mechanosensory neurons enhances sensitivity to vibration, thereby accelerating locomotion. Thus, sensory evoked activity in the RMG circuit arouses locomotion during lethargus through changes in PDF-1 and PDFR-1 signaling. These results raise several interesting questions. Which specific sensory neurons are responsible for arousal? Does the RMG circuit regulate arousal via multiple outputs (i.e. in addition to PDF-1)? Does the RMG circuit function similarly during lethargus and in adults? Is diminished sensory acuity during lethargus required for behavioral quiescence?

Here we show that glutamatergic transmission promotes arousal, we identify glutamatergic neurons and glutamate receptors that mediate arousal, and we show that arousal occurs by distinct mechanisms in lethargus and adult animals.

## Results

### Cholinergic transmission at NMJs is increased in *npr-1* adults

**Fig 1:**
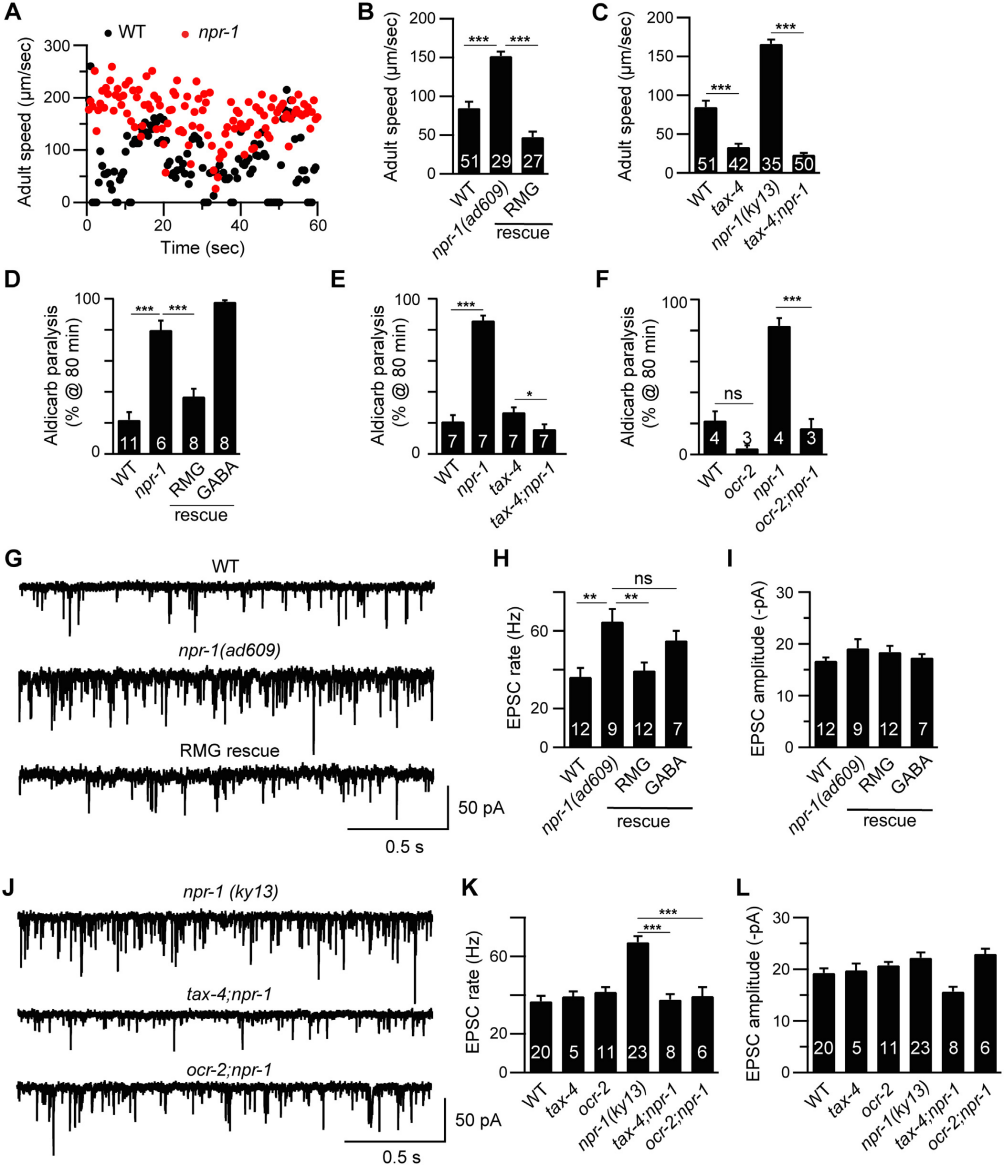
Cholinergic transmission at NMJs is enhanced by increased sensory activity in *npr-1* adults. Locomotion behavior of single adult worms was analyzed for the indicated genotypes. Instantaneous locomotion velocity (A) and average locomotion velocity (B-C) are plotted. (A-C) The *npr-1* adult locomotion defect was rescued by transgenes expressing NPR-1 in the RMG circuit (RMG rescue, *flp-21* promoter), and suppressed in double mutants lacking TAX-4/CNG channels. (D-F) The percentage of animals paralyzed on 1 mM aldicarb at 80 min were plotted for the indicated genotypes. The number of trials is indicated for each genotype. Full time courses of aldicarb-induced paralysis are shown in S2A–S2C Fig. (D) The *npr-1* aldicarb hypersensitivity was rescued by transgenes expressing NPR-1 in the RMG circuit (RMG rescue, *flp-21* promoter) but not by those expressed in GABAergic neurons (GABA rescue, *unc-25* and *unc-30* promoters). (E-F) The *npr-1* aldicarb hypersensitivity was blocked by mutations inactivating TAX-4/CNG channels or OCR-2/TRPV channels. (G-L) mEPSCs were recorded from body wall muscles of adult worms for the indicated genotypes. Representative traces of mEPSCs (G and J) and summary data are shown (H, I, K, and L). (G-I) The *npr-1* cholinergic transmission defect was rescued by transgenes expressing NPR-1 in the RMG circuit (RMG rescue, *flp-21* promoter) but not by those expressed in GABAergic neurons (GABA rescue, *unc-30* promoter). (J-L) The *npr-1* cholinergic transmission defect was abolished by mutations inactivating TAX-4 or OCR-2. The number of animals analyzed is indicated for each genotype. Error bars indicate SEM. Values that differ significantly are indicated (*, *p* <0.05; **, *p* <0.01; ***, *p* <0.001; ns, not significant).

Adult *npr-1* mutants exhibit accelerated locomotion (Fig 1A–1C), as shown in prior studies [21]. Faster adult locomotion suggests that locomotion circuit activity has been altered. Consistent with this idea, *npr-1* mutant adults have enhanced sensitivity to the paralytic effects of a cholinesterase inhibitor (aldicarb) (Fig 1D–1F and S2A Fig) [22], indicating increased excitatory transmission at neuromuscular junctions (NMJs). To more directly assess changes in synaptic transmission, we recorded miniature excitatory post-synaptic currents (mEPSCs) in body muscles, which are evoked by acetylcholine (ACh) release at NMJs. The mEPSC rate observed in *npr-1* adults was significantly higher than in wild type controls while mEPSC amplitudes were unaltered (Fig 1G–1I). Faster mEPSC rates suggest that ACh release from motor neurons was increased whereas unaltered mEPSC amplitudes imply that muscle responsiveness to secreted ACh was unaffected. By contrast, neither ACh release evoked by depolarizing motor neurons with a stimulating electrode (evoked EPSCs), nor transmission at GABAergic NMJs (assessed by miniature inhibitory post-synaptic currents, mIPSCs) was altered in *npr-1* mutants (S1 Fig). This constellation of electrophysiological defects suggests that tonic ACh release (assessed by mEPSC rate) was enhanced in *npr-1* mutants, whereas other forms of neurotransmitter release (evoked ACh release and tonic GABA release) were unaffected. Enhanced tonic ACh release at NMJs could account for the accelerated locomotion rate observed in *npr-1* adults.

### Enhanced cholinergic transmission in *npr-1* adults is caused by increased sensory activity

Prior studies showed that several behavioral phenotypes exhibited by *npr-1* mutants are caused by enhanced sensitivity to environmental cues. In particular, sensory responses mediated by the RMG circuit are enhanced in *npr-1* mutants [14,17,18] and this enhanced sensory acuity is required for accelerated locomotion rates during lethargus [11,20]. We did several experiments to determine if enhanced RMG circuit activity is also required for increased cholinergic transmission in *npr-1* adults. A transgene restoring *npr-1* expression in the RMG circuit (using the *flp-21* promoter) rescued the accelerated locomotion (Fig 1B), enhanced aldicarb sensitivity (Fig 1D and S2A Fig), and faster mEPSC rate (Fig 1G–1I) defects of *npr-1* adults. By contrast, an *npr-1* transgene expressed in GABAergic neurons lacked rescuing activity (Fig 1D–1H). These results indicate that NPR-1 acts in the RMG circuit to slow adult locomotion. Similarly, mutations inactivating ion channels required for sensory transduction (TAX-4/CNG and OCR-2/TRPV) in the RMG circuit suppressed the *npr-1* adult locomotion (Fig 1C), aldicarb sensitivity (Fig 1E and 1F and S2B and S2C Fig), and mEPSC rate (Fig 1J and 1K) defects. Collectively, these results suggest that the accelerated adult locomotion exhibited by *npr-1* mutants is caused by heightened activity in the RMG sensory circuit and, consequently, corresponds to an aroused state.

### Inactivating PDF signaling does not prevent aroused locomotion in *npr-1* adults

**Fig 2:**
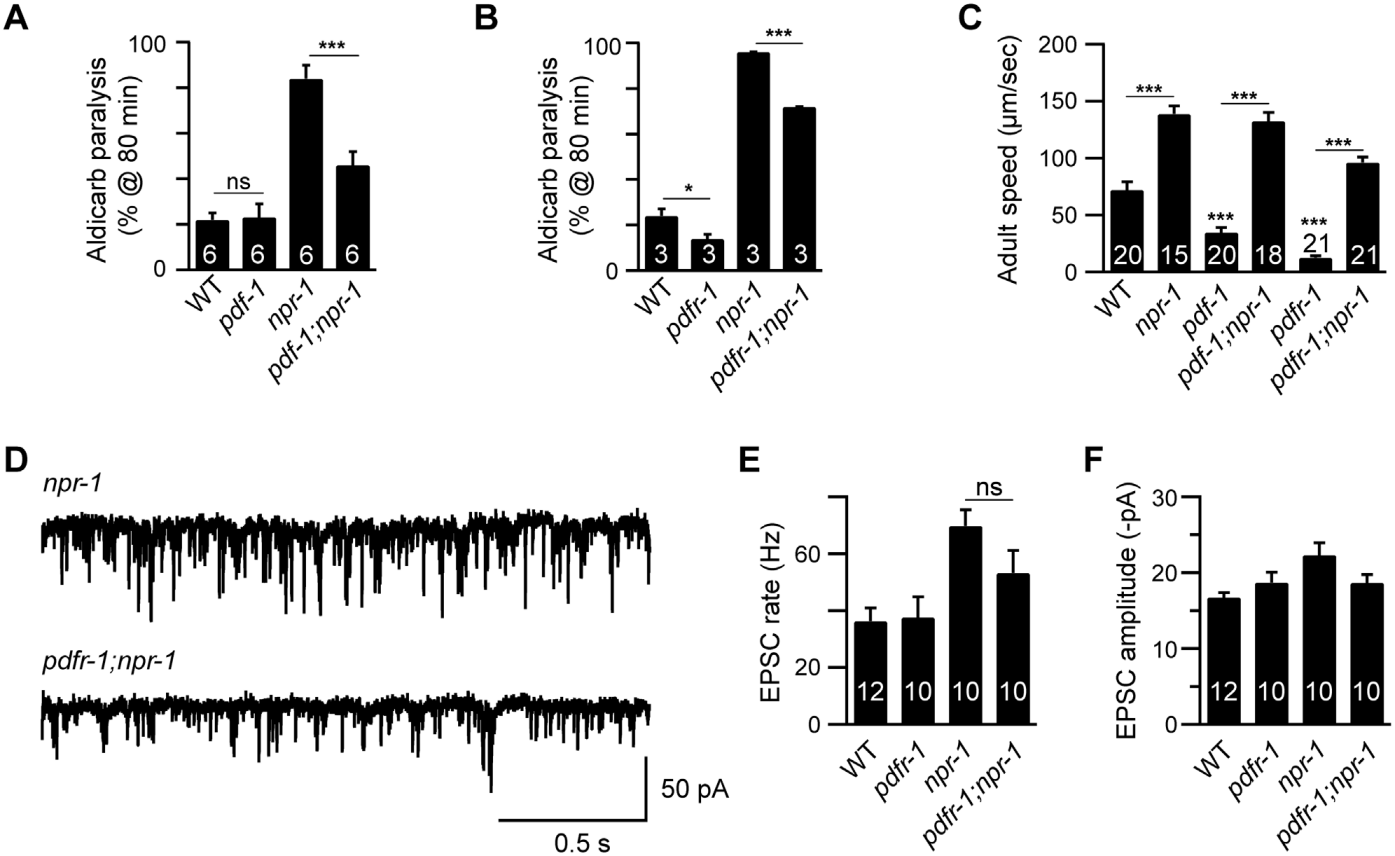
Inactivating PDF signaling does not prevent aroused locomotion in *npr-1* adults. (A-B) The *npr-1* aldicarb hypersensitivity was decreased but not abolished by mutations inactivating PDF-1 or PDFR-1. The percentage of animals paralyzed on 1 mM aldicarb at 80 min were plotted for the indicated genotypes. The number of trials is indicated for each genotype. Full time courses of aldicarb-induced paralysis are shown in S2D and S2E Fig. (C) Locomotion behavior of single adult worms was analyzed for the indicated genotypes. The *npr-1* adult locomotion defect was not blocked by mutations inactivating PDF-1 or PDFR-1. (D-F) mEPSCs were recorded from body wall muscles of adult worms for the indicated genotypes. Representative traces of mEPSCs (D) and summary data are shown (E-F). The *npr-1* cholinergic transmission defect was not suppressed by mutations inactivating PDFR-1. The number of animals analyzed is indicated for each genotype. Error bars indicate SEM. Values that differ significantly are indicated (*, *p*<0.05;**, *p*<0.01;***, *p* <0.001; ns, not significant).

We previously showed that the lethargus quiescence defects exhibited by *npr-1* mutants are caused by increased secretion of Pigment dispersing factor (PDF-1) by cells in the RMG circuit [11]. Because PDF-1 secretion is also increased in *npr-1* adults [11], we tested the idea that the hyperactive adult locomotion of *npr-1* mutants is also caused by increased PDF signaling. Contrary to this idea, we found that *pdf-1* and *pdfr-1* (PDF Receptor-1) mutations reduced but did not eliminate the aldicarb hypersensitivity (Fig 2A and 2B and S2D and S2E Fig), the accelerated locomotion (Fig 2C), and increased mEPSC rate (Fig 2D and 2E) defects of *npr-1* adults. Collectively, these results suggest that additional excitatory outputs from the RMG circuit (i.e. beyond PDF-1) must contribute to the aroused locomotion of *npr-1* adults.

### Glutamate released by sensory neurons is required for *npr-1* locomotion and EPSC defects

**Fig 3:**
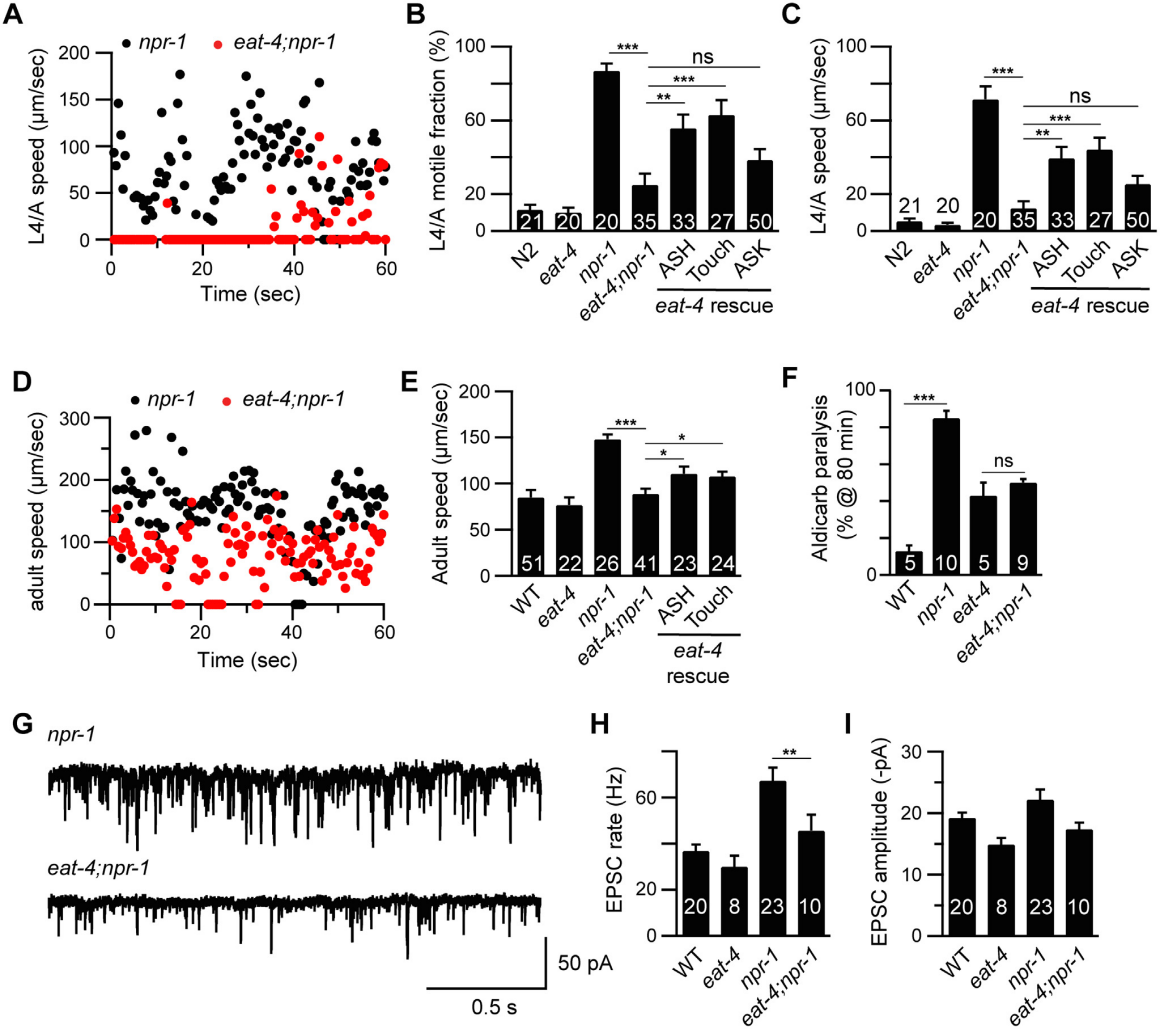
Glutamate released by sensory neurons is required for the *npr-1* locomotion and the cholinergic transmission defects. Locomotion behavior of single worms during the L4/A lethargus (A-C) and in adults (D-E) was analyzed in the indicated genotypes. Instantaneous locomotion velocity (A, D), average motile fraction (B), and average locomotion velocity (C, E) are plotted. The *npr-1* locomotion defect was suppressed by mutations inactivating EAT-4/VGLUT, and partially reinstated by transgenes expressing EAT-4 in ASH neurons (*sra-6* promoter) and touch neurons (*mec-4* promoter) in *eat-4;npr-1* double mutants using the indicated promoters. An EAT-4 transgene expressed in ASK neurons (*sra-9* promoter) lacked rescuing activity. (F) The *npr-1* aldicarb hypersensitivity was suppressed by mutations inactivating EAT-4/VGLUT. The percentage of animals paralyzed on 1 mM aldicarb at 80 min were plotted for the indicated genotypes. The number of trials is indicated for each genotype. Full time courses of aldicarb-inuced paralysis are shown in S2F Fig. (G-I) The *npr-1* cholinergic transmission defect was abolished by mutations inactivating EAT-4/VGLUT. mEPSCs were recorded from body wall muscles of adult worms for the indicated genotypes. Representative traces of mEPSCs (G) and summary data are shown (H-I). The number of animals analyzed is indicated for each genotype. Error bars indicate SEM. Values that differ significantly are indicated (*, *p* <0.05; **, *p* <0.01; ***, *p* <0.001; ns, not significant).

Many *C*. *elegans* sensory neurons are glutamatergic, including two neurons in the RMG circuit (ASH and ASK) and the body touch neurons [23]. To determine if glutamate release by sensory neurons is required for accelerated locomotion in *npr-1* mutants, we analyzed mutations that inactivate the vesicular glutamate transporter (*eat-4* VGLUT), which is primarily expressed in sensory neurons [23]. *eat-4* VGLUT mutations blocked the increased motile fraction and locomotion speed of *npr-1* mutants both during the L4-Adult (L4/A) molt (Fig 3A–3C) and in adults (Fig 3D and 3E). *eat-4* mutations also blocked the hypersensitivity to aldicarb (Fig 3F and S2F Fig) and increased mEPSC rate (Fig 3G and 3H) defects of *npr-1* adults. Transgenes restoring EAT-4 expression in touch neurons and ASH neurons partially reinstated both lethargus (Fig 3B and 3C) and adult locomotion (Fig 3D and 3E) defects in *eat-4*; *npr-1* double mutants, whereas transgenes expressed in ASK lacked rescuing activity (Fig 3B and 3C). *eat-4* transgenes had no effect on lethargus quiescence in wild type animals (S3 Fig). These results suggest that glutamate released by ASH and touch neurons arouses locomotion in L4/A and adult *npr-1* mutants.

### ASH activity is associated with locomotion arousal

**Fig 4:**
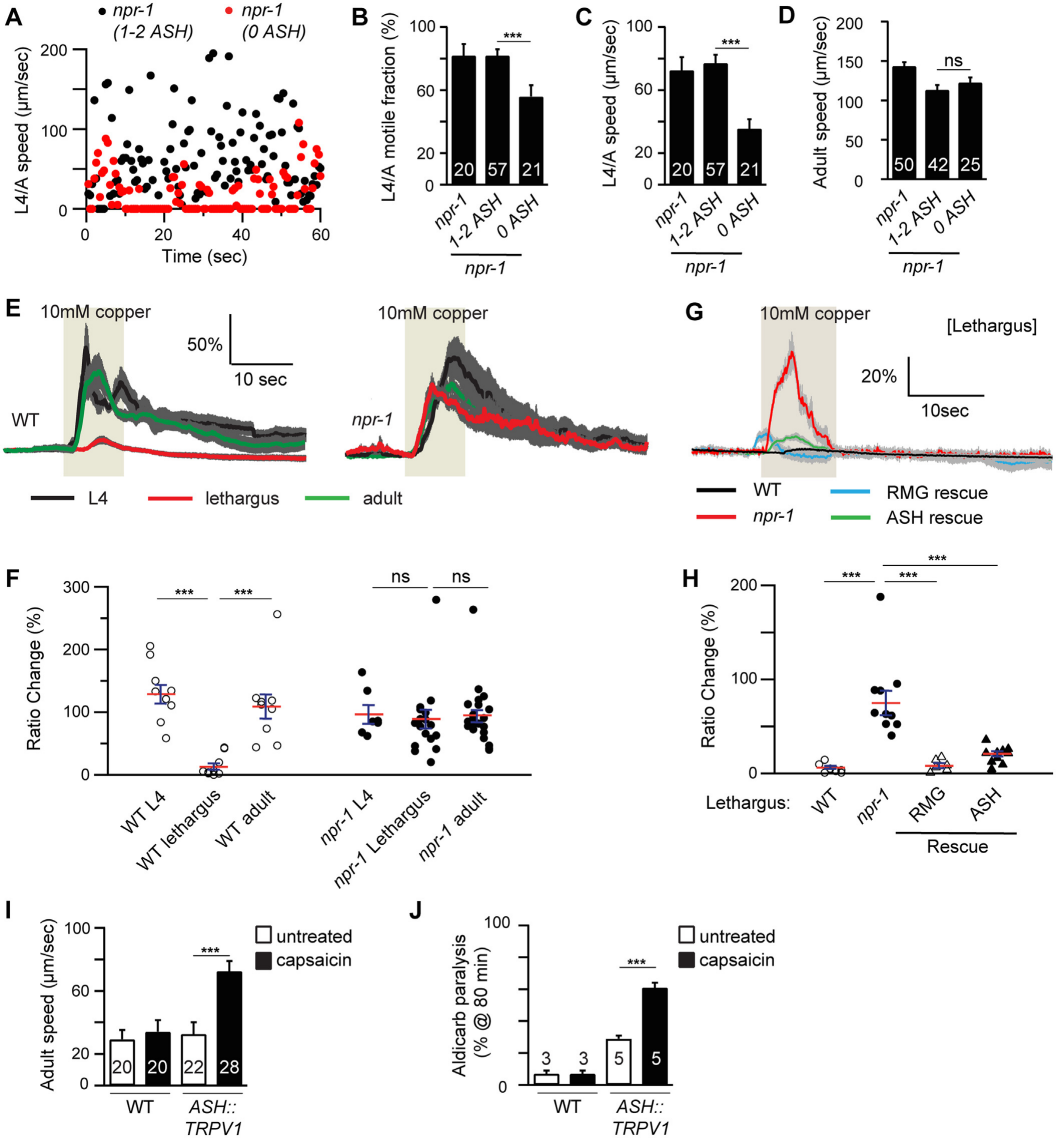
ASH activity is associated with locomotion arousal. Locomotion behavior during the L4/A lethargus (A-C) and in adults (D) of single worms whose ASH neurons were ablated by transgenic overexpression of CED-3 in ASH neurons (*sra-6* promoter) was analyzed in the indicated genotypes. Animals were analyzed by fluorescence microscopy after locomotion recordings to determine if ASH neurons were ablated (1–2 ASH: animals with 1 or 2 ASH intact neurons; 0 ASH: animals lacking viable ASH neurons). Instantaneous locomotion velocity (A), average motile fraction (B), and average locomotion velocity (C-D) are plotted. The *npr-1* locomotion defect during the L4/A lethargus, but not in adults, was partially suppressed in the transgenic animals in which both of ASH neurons were ablated (0 ASH). (E-H) Copper-evoked calcium transients in ASH were analyzed in L4, L4/A, and adults of the indicated genotypes using cameleon as a calcium indicator. Averaged responses (E, G), and the amplitudes of individual trials (F, H) are shown for each genotype. Each trace represents the average percentage change in YFP/CFP fluorescence ratio. The light tan rectangle indicates the duration for which 10 mM copper was applied. Dark gray shading of each trace indicates SEM of the mean response. (E-F) Copper-evoked calcium transients in ASH neurons were significantly reduced during L4/A lethargus, and this effect was abolished in *npr-1* mutants. (G-H) This defect during L4/A lethargus was rescued by transgenes expressing NPR-1 in the RMG circuit (RMG rescue, *flp-21 promoter*) or in ASH neurons (ASH rescue, *sra-6 promoter*). (I-J) Forced depolarization of ASH neurons increased adult locomotion velocity (I) and aldicarb sensitivity (J). Rat TRPV1 was ectopically expressed in ASH neurons (using the *sra-6* promoter). (I) Locomotion behavior of adult transgenic worms was analyzed with or without capsaicin treatment (5 hours). Average locomotion velocity (I) is plotted. Capsaicin treatment increased adult locomotion velocity in transgenic animals expressing TRPV1 in ASH neurons, but not in wild type controls. The number of animals analyzed is indicated for each genotype. (J) The percentage of animals paralyzed on 1 mM aldicarb at 80 min with or without capsaicin treatment (2–3 hours pretreatment) were plotted for the indicated genotypes. The number of trials is indicated for each genotype. Full time courses for aldicarb-induced paralysis are shown in S2G Fig. Capsaicin treatment increased aldicarb sensitivity in transgenic animals expressing TRPV1 in ASH neurons, but not in wild type controls. Error bars indicate SEM. Values that differ significantly are indicated (***, *p* <0.001; ns, not significant).

The preceding results suggest that ASH synaptic output arouses locomotion in *npr-1* mutants. We did several additional experiments to test this idea. If altered ASH output were required for aroused locomotion, we would expect that *npr-1* mutants lacking ASH neurons would have increased locomotion quiescence. To test this idea, we induced ASH cell death with a transgene that expresses the pro-apoptotic caspase CED-3. Killing ASH significantly decreased the L4/A motile fraction and locomotion rate in *npr-1* mutants (Fig 4A–4C). By contrast, ASH ablation had little effect on the locomotion rate of *npr-1* adults (Fig 4D).

To determine if ASH activity is increased in *npr-1* mutants during lethargus, we examined sensory-evoked calcium responses in ASH, using the genetically encoded calcium indicator Cameleon. ASH mediates avoidance responses to copper and hyper-osmotic stimuli. Consistent with a recent study [12], the magnitude of copper (Fig 4E and 4F) and glycerol-evoked (S4A and S4B Fig) calcium transients in ASH was significantly decreased during lethargus in wild-type animals. Decreased ASH responsiveness to copper and glycerol during L4/A lethargus was blocked in *npr-1* mutants, whereas ASH responsiveness in adults was unaltered in *npr-1* mutants (Fig 4E and 4F and S4A and S4B Fig). Transgenes expressing NPR-1 in the RMG circuit (using the *flp-21* promoter) or in ASH (using the *sra-6* promoter) reinstated the L4/A decrease in copper and glycerol-evoked ASH calcium transients in *npr-1* mutants (Fig 4G and 4H and S4C and S4D Fig). These results suggest that NPR-1 acts in ASH to inhibit sensory responses and that increased ASH activity is required for accelerated locomotion of *npr-1* mutants during lethargus but not in adults.

**Fig 5:**
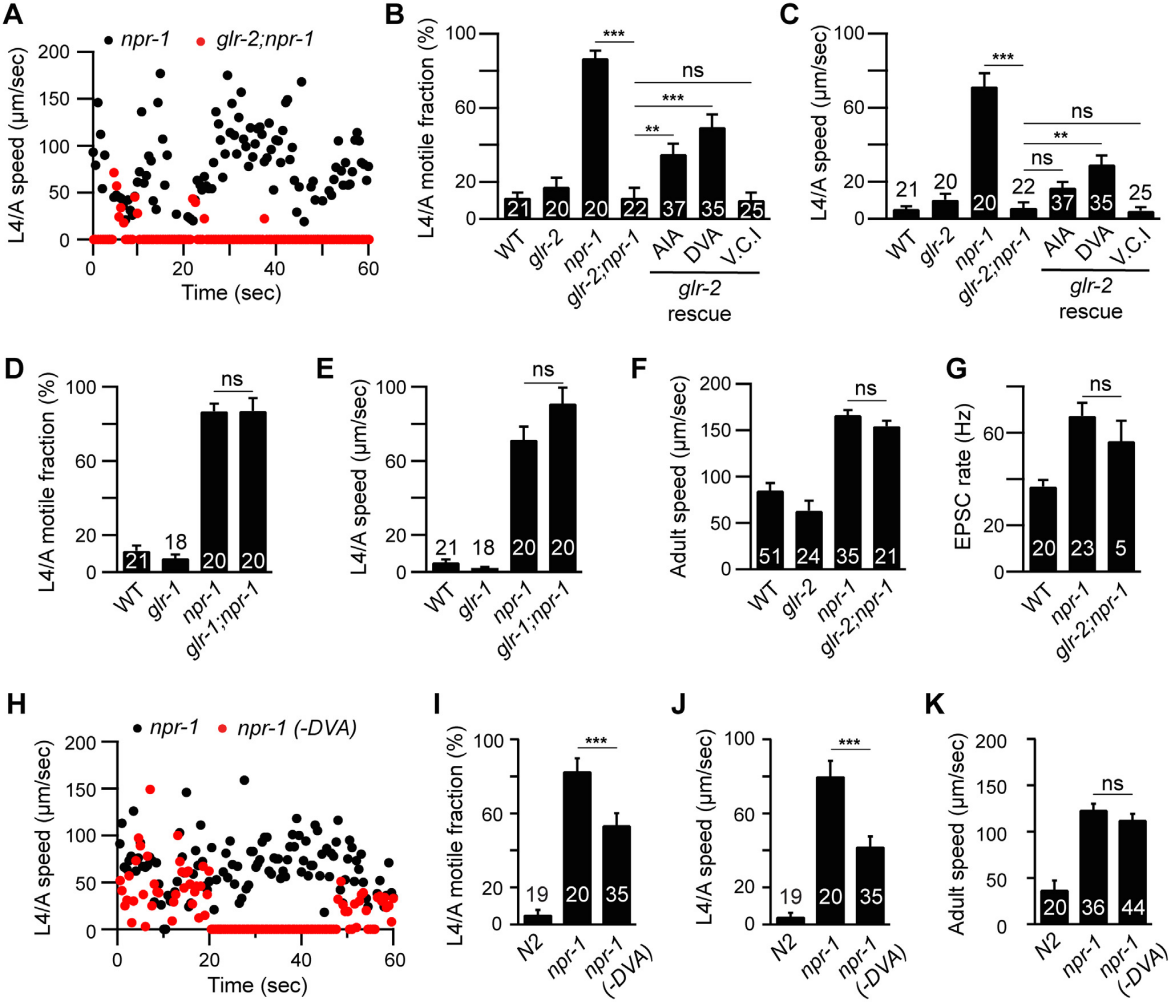
GLR-2 AMPA receptors are required for the *npr-1* lethargus defect. Locomotion behavior of single worms during the L4/A lethargus (A-E and H-J) and in adults (F, K) was analyzed in the indicated genotypes. Instantaneous locomotion velocity (A, H), average motile fraction (B, D, and I), and average locomotion velocity (C, E, F, J and K) are plotted. (A-C) The *npr-1* locomotion defect during L4/A lethargus was suppressed by mutations inactivating *glr-2* AMPA receptors, and partially reinstated by transgenes expressing GLR-2 in AIA (*gcy-28(d)* promoter) and DVA (*nlp-12* promoter) neurons, but not in Ventral Cord Interneurons (V.C.I., *glr-1* promoter) in *glr-2;npr-1* double mutants using the indicated promoters. (D-E) *glr-1* mutations had no suppressing effect. (F) *glr-2* mutations did not block the increased locomotion in *npr-1* adults. (G) mEPSCs were recorded from body wall muscles of the adult worms for the indicated genotypes. Summary data are shown. (G) *glr-2* mutations did not block the increased mEPSC rate in *npr-1* adults. (H-K) Locomotion behavior during the L4/A lethargus (H-J) and in adults (K) of single worms whose DVA neuron is ablated by transgenic overexpression of CED-3 in DVA neuron (*nlp-12* promoter) was analyzed in the indicated genotypes. Animals were analyzed by fluorescence microscopy after locomotion recordings to determine if DVA was ablated. The *npr-1* locomotion defect during the L4/A lethargus, but not in adults, was partially suppressed in the transgenic animals in which DVA was ablated (-DVA). The number of animals analyzed is indicated for each genotype. Error bars indicate SEM. Values that differ significantly are indicated (**, *p* <0.01; ***, *p* <0.001; ns, not significant).

To determine if increased ASH activity is sufficient to arouse locomotion, we analyzed locomotion after artificially depolarizing ASH neurons. For this experiment, we utilized transgenic animals that express rat TRPV1 capsaicin receptors in ASH neurons [24]. In these animals, capsaicin treatment evokes ASH-mediated avoidance behaviors [24]. A 5-hour capsaicin treatment had little effect on L4/A motile fraction and locomotion velocity [11], whereas capsaicin treatment significantly accelerated adult locomotion and increased aldicarb sensitivity (Figs 4I–5J and S2G Fig). These effects were not observed in animals lacking TRPV1 expression in ASH neurons (Fig 4I and 4J). Thus, forced ASH depolarization was sufficient to arouse adult but not lethargus locomotion. Collectively, these results suggest that diminished and heightened ASH activity is associated with locomotion quiescence and arousal respectively; however, the magnitude of ASH’s arousing effects differ between lethargus and adult animals.

### GLR-2 AMPA receptors are required for the *npr-1* lethargus defect

Which glutamate receptors arouse locomotion in *npr-1* mutants? Glutamate-activated cation channels, AMPA (GLR-1 and -2) and NMDA (NMR-1 and -2) receptors, mediate excitatory transmission at ASH-interneuron [25–27]. The *npr-1* L4/A quiescence defect was abolished in *glr-2; npr-1* double mutants (Fig 5A–5C), while *glr-1* mutations had no effect (Fig 5D and 5E). By contrast, *glr-1*, *glr-2*, and *nmr-1* mutations had little effect on *npr-1* adult locomotion (Fig 5F and S5 Fig). Similarly, *glr-2* mutations did not block the increased mEPSC rate in *npr-1* adults (Fig 5G). These results suggest that GLR-2 AMPA receptors are specifically required for the aroused locomotion during the L4/A lethargus in *npr-1* mutants.

### GLR-2 AMPA receptors act in AIA and DVA to mediate arousal

Which synaptic targets of ASH and touch neurons mediate locomotion arousal? To address this question, we identified the neurons in which GLR-2 function is required. Aroused L4/A locomotion requires GLR-2 but not GLR-1 receptors; consequently, we reasoned that the relevant neurons are likely to express GLR-2 but not GLR-1. GLR-1 and GLR-2 are co-expressed in many neurons; however, a few GLR-2-expressing neurons lack GLR-1, including DVA (a stretch-activated neuron) and AIA (an interneuron in the head ganglia) [25–27]. The L4/A quiescence defect was partially restored in *glr-2; npr-1* double mutants by transgenes expressing GLR-2 in DVA and AIA neurons, whereas transgenes expressed in the ventral cord interneurons (using the *glr-1* promoter) failed to rescue (Fig 5B and 5C). Transgenic expression of GLR-2 in DVA or AIA had no effect on lethargus quiescence in wild type worms (S3 Sig). These results suggest that GLR-2 AMPA receptors expressed in AIA and DVA neurons arouse L4/A locomotion in *npr-1* mutants. DVA receives direct synaptic input from the touch neuron PLM while AIA receives direct input from ASH [28]. Thus, increased transmission at ASH-AIA and PLM-DVA synapses could account for GLR-2’s effects on locomotion rate. Because we only observed partial rescue by *glr-2* transgenes expressed in AIA and DVA, it is likely the GLR-2 function is required in additional (as yet unidentified) neurons.

**Fig 6:**
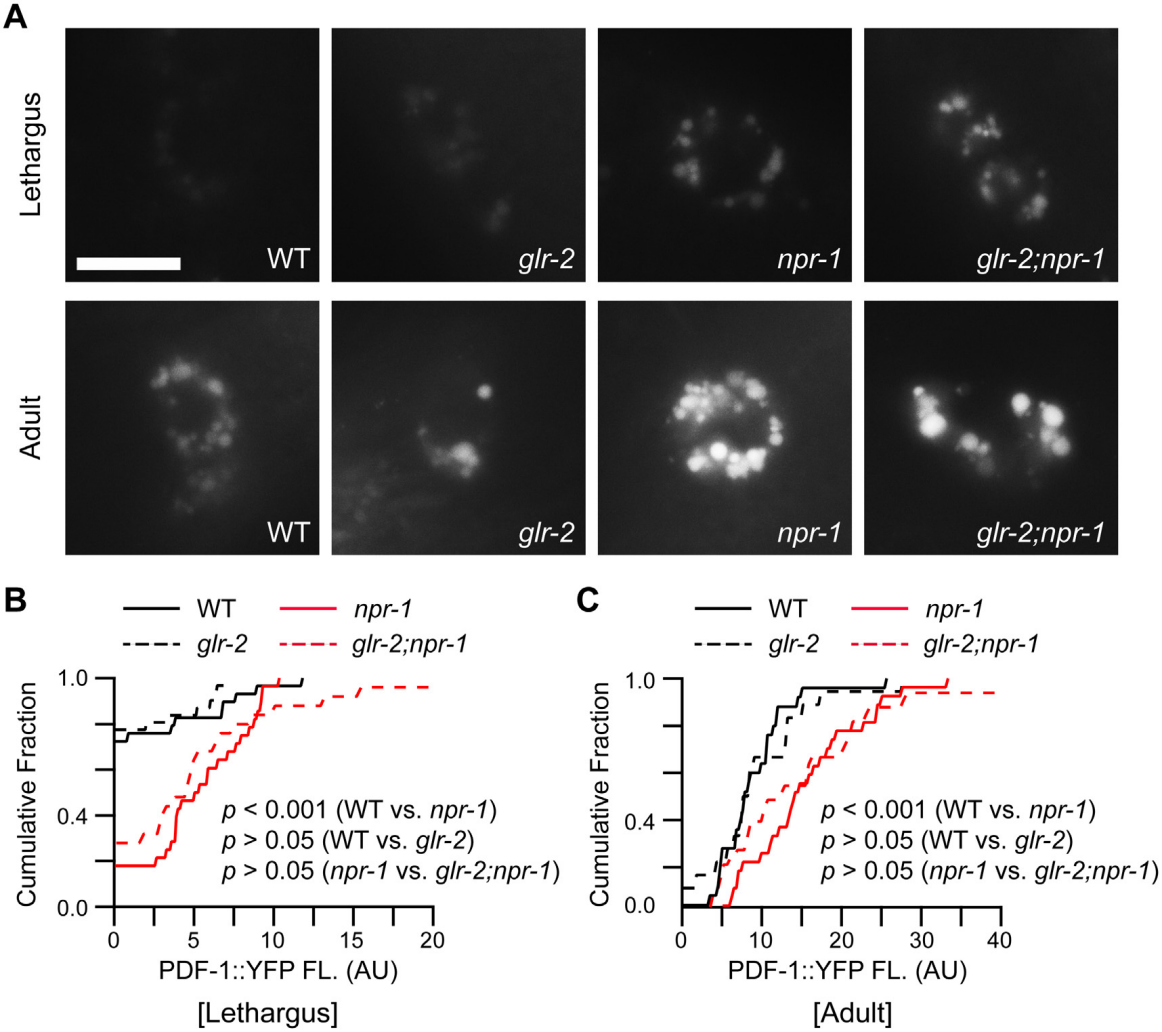
PDF-1 secretion is not altered in *glr-2* mutants. PDF-1 secretion was analyzed in the indicated genotypes. YFP-tagged PDF-1 was expressed with the *pdf-1* promoter. Representative images (A) and summary data (cumulative fraction) (B-C) are shown for coelomocyte fluorescence in L4/A lethargus and 1-day old adults of the indicated genotypes. PDF-1::YFP coelomocyte fluorescence was dramatically increased in *npr-1* mutants during the L4/A lethargus and in adults as previously reported [11]. Mutations inactivating GLR-2 did not alter PDF-1::YFP coelomocyte fluorescence during L4/A lethargus (B) and in adults (C) in either wild type or *npr-1* mutants. Scale bar indicates 10 μm. *p* values are indicated for each comparison (Kolmogorov-Smirnov test).

How do AIA and DVA arouse locomotion? AIA neurons provide synaptic input to ASK and ASI, both of which express PDF-1 [11,29]. Thus, heightened AIA activity could arouse locomotion by enhancing PDF-1 secretion. To assess the level of PDF-1 secretion, we analyzed PDF-1::YFP fluorescence in the endolysosomal compartment of coelomocytes, which are specialized scavenger cells that internalize proteins secreted into the body cavity [30,31]. Inactivating GLR-2 did not alter PDF-1::YFP fluorescence in coelomocytes in both adult and L4/A animals (Fig 6). These results suggest that the arousing effects of GLR-2 are not mediated by changes in PDF secretion. DVA neurons receive direct synaptic input from the PLM touch neurons [32], and secrete NLP-12 (a neuropeptide that accelerates locomotion) [33]. Thus, increased DVA activity could contribute to locomotion arousal in *npr-1* mutants. Three results support this idea. First, PLM neurons exhibit enhanced touch-evoked calcium responses in adult *npr-1* mutants (S6 Fig). Thus, PLM neurons have increased sensory acuity in *npr-1* mutants, similar to the effect we previously showed for ALM neurons [11]. Second, inducing DVA cell death (with a CED-3 transgene) significantly reduced *npr-1* locomotion rate during L4/A lethargus (Fig 5H–5J), but not in adults (Fig 5K). Third, DVA secretion of NLP-12 is significantly increased in *npr-1* mutants [33], indicating increased DVA activity. These results suggest that PLM neurons provide enhanced excitatory input to DVA in *npr-1* mutants, which promotes aroused L4/A locomotion.

## Discussion

To investigate the circuit mechanisms for arousal, we analyzed the locomotion of *npr-1* mutants in awake (adult) and quiescent (lethargus) states. Our results lead to five conclusions. First, multiple classes of sensory neurons contribute to arousal. Second, diminished sensory acuity is a circuit mechanism for promoting behavioral quiescence. Third, glutamate and neuropeptides are utilized as excitatory outputs from sensory neurons to arouse locomotion. Fourth, different mechanisms are utilized to arouse locomotion at different times during development. And fifth, we provide further evidence that arousal mechanisms are conserved across phylogeny.

### A broad network of sensory neurons contribute to arousal

Multiple classes of sensory neurons arouse locomotion during lethargus and in adults, including: mechanosensory neurons (ALM and PLM), a nociceptive neuron (ASH), a pheromone sensing neuron (ASK), and a stretch sensing neuron (DVA). Lethargus quiescence is accompanied by diminished sensory-evoked responses in ALM, PLM, and ASH (this study and [11–13]). PDF-1 secretion from ASK neurons is significantly reduced during lethargus, implying that ASK neurons also have diminished activity during lethargus [11]. *npr-1* mutations prevent the dampened ALM (mechanosensory) and ASH (nociceptive) responses during lethargus and this was accompanied by decreased locomotion quiescence (this study and [11]). The arousing effects of *npr-1* mutations are blocked (or diminished) by mutations that decrease sensory responsiveness (e.g. *tax-4* CNG and *osm-9* TRPV mutations) [11], or by ablating sensory neurons (e.g. ASH and DVA). Forced activation of ASH neurons arouses adult locomotion. Collectively, these results imply that a broad network of sensory neurons arouses locomotion, which allows *C*. *elegans* to adapt its behavior across a broad range of developmental and physiological circumstances.

### Sensory gain control as a mechanism for producing quiescence and arousal

NPR-1 promotes behavioral quiescence by diminishing the sensitivity of many sensory modalities. NPR-1 directly inhibits ASH responses and indirectly inhibits other sensory neurons (ALM, PLM, and DVA) via decreased glutamate and neuropeptide release. Thus, gating of sensory perception by NPR-1 provides a circuit mechanism for producing aroused and quiescent locomotion in *C*. *elegans*.

Our results do not exclude the possibility that additional mechanisms (beyond sensory gating by NPR-1) contribute to arousal and quiescence. Both quiescence (during lethargus) and arousal (following molts) persist in microfluidic chambers where many sensory cues are minimized [19]. In particular, oxygen tension is likely to be very low in these chambers, which would greatly diminish NPR-1’s effects on behavior [15,16]. Thus, the quiescence and arousal exhibited in microfluidic chambers implies that additional mechanisms beyond NPR-1 must contribute to expressing these behavioral states. It will be interesting to determine if these NPR-1 independent mechanisms also act by gating sensory activity.

### Sensory-evoked glutamate and neuropeptide release arouses locomotion

Sensory neurons release glutamate and/or neuropeptides in response to external cues, which then engage downstream motor circuits in behavioral outputs. Our prior study shows that sensory-evoked PDF-1 secretion promotes locomotion arousal by enhancing touch neuron responsiveness. Neuropeptides also mediate arousal in flies (PDF) [34], fish and mammals (orexin/hypocretin) [35,36].

Here we show that sensory evoked glutamate release also plays a role in arousal. Mutations inactivating the EAT-4/VGLUT decreased locomotion arousal in lethargus and in adults. EAT-4 is almost exclusively expressed in sensory neurons [23] and transgenes restoring EAT-4 in touch neurons and ASH neurons re-instates locomotion arousal in *npr-1* mutants. These results suggest that sensory neurons utilize both glutamate and neuropeptides as excitatory outputs to arouse locomotion.

Our results suggest that exaggerated glutamate release at ASH-AIA and PLM-DVA synapses arouses locomotion during lethargus in *npr-1* mutants. ASH and PLM neurons have enhanced sensory evoked activity in *npr-1* mutants, which is expected to produce enhanced glutamate release at ASH-AIA and PLM-DVA synapses. GLR-2 receptors are expressed in AIA and DVA. *glr-2* mutations block the aroused L4/A locomotion of *npr-1* mutants and arousal is re-instated by transgenes expressing GLR-2 in AIA and DVA. Finally, calcium responses in AIA [14], and neuropeptide secretion from DVA [33] are both enhanced in *npr-1* mutants, indicating that these neurons have increased activity. We observed only partial rescue of aroused locomotion by transgenes restoring EAT-4 expression in ASH and touch neurons or by those expressing GLR-2 in AIA or DVA; consequently, it is likely that glutamate released by other sensory neurons also contributes to the aroused L4/A locomotion in *npr-1* mutants.

Much less is known about the role of glutamate in arousal in other systems. Glutamate release has widespread effects throughout the brain in mammals, which complicates the analysis of its effects on arousal. Microinjection of glutamate or AMPA into lateral hypothalamic area increased locomotor activity and duration of waking episodes in rodents [37,38], while microdialysis of CNQX, an AMPA receptor antagonist, into the thalamus promotes sleep in cats [39]. Glutamate also induces fictive locomotion in lamprey [40]. In these cases, however, the circuit mechanisms underlying glutamate’s arousing effects are not known.

### Comparing lethargus and adult arousal mechanisms

Mutants lacking NPR-1 exhibit accelerated locomotion in adults and during lethargus [11,18]. Several results suggest that locomotion arousal in adult and lethargus is established by a shared central sensory circuit. First, in both adult and lethargus, enhanced activity in the RMG sensory circuit accelerates locomotion, whereas decreased sensory transduction in the RMG circuit (i.e. by inactivating TAX-4 or OSM-9) abolishes *npr-1’s* hyperactive locomotion defect [11,14], suggesting that the RMG circuit activity stimulates arousal in both awake and quiescent states. Second, EAT-4 acts in ASH and touch neurons to mediate hyperactive locomotion of *npr-1* adult and lethargus stage animals, suggesting that glutamate release from these sensory neurons is required for locomotion arousal in *npr-1* mutants.

On the other hand, several results suggest that the mechanisms that arouse locomotion differ between adult and lethargus animals. Inactivating GLR-2 AMPA receptors blocks the hyperactive locomotion of *npr-1* mutants during lethargus but not in adults. Aroused locomotion in *npr-1* adults persists in *glr-1*, *glr-2*, and *nmr-1* mutants, indicating that other glutamate receptors are responsible for arousing adult locomotion. Similarly, artificial activation of ASH accelerates adult but not lethargus locomotion. Collectively, our results suggest that multiple sensory circuits govern locomotion arousal throughout development but that the relative contribution of each circuit to arousal differs depending on the developmental stage.

## Materials and Methods

### Strains

Strain maintenance and genetic manipulation were performed as described [41]. Animals were cultivated at 20°C on agar nematode growth media (NGM) seeded with OP50 (for imaging and behavior) or HB101 *E*.*coli* (for electrophysiology). Wild type reference strain was N2 Bristol. Strains used in this study are as follows:

### Mutant strains and integrants

KP6048 *npr-1(ky13) X*

DA609 *npr-1(ad609) X*

KP6064 *npr-1(ok1447) X*

PR678 *tax-4(p678) III*

CX4544 *ocr-2(ak47) IV*

LSC27 *pdf-1(tm1996) III*

KP6340 *pdfr-1(ok3425) III*

MT6308 *eat-4(ky5) III*

KP0004 *glr-1(n2461) III*

VM487 *nmr-1(ak4) II*

KP6057 *ocr-2(ak47) IV;npr-1(ok1447) X*

KP6058 *ocr-2(ak47) IV;npr-1(ky13) X*

KP6060 *tax-4(p678) III;npr-1(ky13) X*

KP6061 *tax-4(p678) III;npr-1(ok1447) X*

KP6100 *pdf-1(tm1996) III;npr-1(ky13) X*

KP6410 *pdfr-1(ok3425) III;npr-1(ky13) X*

KP6349 *eat-4(ky5) III; npr-1(ky13) X*

CX4978 *kyIs200[sra-6p*::*VR1*, *elt-2p*::*NLS-gfp] (Gift from Cori Bargmann)*

KP6414 *nmr-1(ak4) II; npr-1(ky13) X*

KP6415 *glr-1(n2461) III;npr-1(ky13) X*

VM1123 *dpy-19(n1347) glr-2(ak10) III*

KP6740 *dpy-19(n1347) glr-2(ak10) III; npr-1(ky13) X*

KP7362 npr-1(ky13) X; nuIs439[nlp-12p::GFP]; nuIs519[nlp-12p::ced-3::GFP, vha-6::mCherry]

KP6693 *nuIs472 [pdf-1p*::*pdf-1*::*venus*, *vha-6p*::*mCherry]*

KP6743 *npr-1(ky13) X; nuIs472*

KP7194 *dpy-19(n1347) glr-2(ak10) III; nuIs472*

KP7195 *dpy-19(n1347) glr-2(ak10) III; npr-1(ky13) X; nuIs472*

AQ906 *bzIs17[mec-4p*::*YC2*.*12]*

KP6681 *npr-1(ky13) X; bzIS17*

### Strains containing extrachromosomal arrays

CX9396 *npr-1(ad609) X;kyEx1966[flp-21p*::*npr-1 SL2 GFP*, *ofm-1p*::*dsRed] (Gift from Cori Bargmann)*

KP6051 *npr-1(ad609) X;nuEx1519[unc-25p*::*npr-1*::*gfp*, *myo-2p*::*NLS-mCherry]*

KP6053 *npr-1(ad609) X;nuEx1520[unc-30p*::*npr-1*::*gfp*, *myo-2p*::*NLS-mCherry]*

KP7149, KP7150 *eat-4(ky5) III; npr-1(ky13) X; nuEx1613-1614[sra-6p*::*eat-4*, *myo-2p*::*NLS-mCherry]*

KP7176, KP7177 *eat-4(ky5) III; npr-1(ky13) X; nuEx1615-1616[sra-9p*::*eat-4*, *vha-6p*::*mCherry]*

KP7198, KP7199 eat-4(ky5) III; npr-1(ky13) X; nuEx1640-1641[mec-4p::eat-4, vha-6p::mCherry]

KP7442 npr-1(ky13) X; nuEx1684[sra-6p::ced-3::GFP, sra-6p::mCherry, vha-6p::mCherry]

KP7633 nuEx1613[sra-6p::eat-4, myo-2p::NLS-mCherry]

KP7634 nuEx1640[mec-4p::eat-4, vha-6p::mCherry]

AQ3304 *ljEx239[sra-6*::*YC*.*360]*

KP7353 npr-1(ky13) X; ljEx239

KP7443 npr-1(ky13) X; ljEx239; nuEX1607[flp-21p::npr-1, myo-2p::NLS-mCherry]

KP7495 npr-1(ky13) X; ljEx239; nuEX1683[sra-6p::npr-1, vha-6p::mCherry]

KP7191 dpy-19(n1347) glr-2(ak10) III; npr-1(ky13) X; nuEx1637[nlp-12p::glr-2(gDNA),myo-2p::NLS-mCherry]

KP7192 dpy-19(n1347) glr-2(ak10) III; npr-1(ky13) X; nuEx1638[gcy-28(d)p::glr-2(gDNA),vha-6p::mCherry]

KP7354, KP7355, KP7356 dpy-19(n1347) glr-2(ak10) III; npr-1(ky13) X; nuEx1642-1644[glr-1p::glr-2(gDNA), vha-6p::mCherry]

KP7635 nuEx1637[nlp-12p::glr-2(gDNA),myo-2p::NLS-mCherry]

KP7636 nuEx1638[gcy-28(d)p::glr-2(gDNA),vha-6p::mCherry]

### Constructs

#### *eat-4* rescue constructs *(sra-6p*::*eat-4* (KP#2204), *sra-9p*::*eat-4* (KP#2205), and *mec-4p*::*eat-4* (KP#2207)

*eat-4* cDNA was amplified by PCR and ligated into expression vectors (pPD49.26) containing the *sra-6* (~3.8kb 5’ regulatory sequence: ASH expression), *sra-9* (~3kb 5’ regulatory sequence: ASK expression), or *mec-4* (~1.1kb 5’ regulatory sequence: Touch neuron expression) promoters.

#### glr-2 rescue constructs (nlp-12p::glr-2 (KP#2211), gcy-28(d)p::glr-2 (KP#2209), and glr-1p::glr-1 (KP#2208)

*glr-2* genomic DNA was amplified by PCR and ligated into expression vectors (pPD49.26) containing the *nlp-12* (~400 bp 5’ regulatory sequence: DVA expression), *gcy-28(d)* (~2,9kb 5’ regulatory sequence: AIA expression), or *glr-1* (~5.3kb 5’ regulatory sequence: ventral cord interneuron (VCI) expression) promoters.

#### Cell ablation constructs *(sra-6p*::*ced-3*::*GFP* (KP#2151) and *nlp-12p*::*ced-3*::*GFP* (KP#2302)

*ced-3* genomic DNA and GFP were amplified by overlapping PCR and ligated into expression vectors (pPD49.26) (using NheI and SacI restriction sites) containing the *sra-6* (~3.8 kb 5’ regulatory sequence: ASH expression) or *nlp-12* (~400 bp 5’ regulatory sequence: DVA expression) promoters.

### Transgenes and germline transformation

Transgenic strains were generated by microinjection of various plasmids with coinjection markers (*myo-2p*::*NLS-mCherry* (KP#1480) and *vha-6p*::*mcherry* (KP#1874)). Injection concentration was 40–50 ng/μl for all the expression constructs and 10 ng/μl for coinjection markers. The empty vector *pBluescript* was used to bring the final DNA concentration to 100 ng/μl. The *flp-21* promoter (which is expressed in the RMG, ASH, ADL, ASK, URX, and ASI neurons [14]) was used to express transgenes in the RMG circuit.

### Lethargus locomotion and behavior analysis

Lethargus locomotion was analyzed as previously described [11]. Well-fed late L4 animals were transferred to full lawn OP50 bacterial plates. After 1 hour, locomotion of animals in lethargus (determined by absence of pharyngeal pumping) was recorded on a Zeiss Discovery Stereomicroscope using Axiovision software. Locomotion was recorded at 2 Hz for 60 seconds. Centroid velocity of each animal was analyzed at each frame using object-tracking software in Axiovision. Motile fraction of each animal was calculated by dividing the number of frames with positive velocity value with total number of frames. Speed of each animal was calculated by averaging the velocity value at each frame. Quantitative analysis was done using a custom written MATLAB program (Mathworks). Statistical significance was determined using one-way ANOVA with Tukey test for multiple comparisons and two-tailed Student’s t test for pairwise comparison.

### Adult locomotion and behavior analysis

Locomotion of adult animals was analyzed with the same setup as lethargus locomotion analysis described above, except that well-fed adult animals were monitored 1–1.5hr after the transfer to full lawn OP50 bacterial plates. For the capsaicin treatment (Fig 4I), 1 day-old animals were transferred to NGM plates containing 50 μM capsaicin (with food), treated with capsaicin for 5 hours, and recorded for their locomotion. Statistical significance was determined using one-way ANOVA with Tukey test for multiple comparisons and two-tailed Student’s t test for pairwise comparison.

### Cell ablations

Neurons were ablated in *npr-1(ky13)* mutant worms by transgenes co-expressing CED-3 and a fluorescent protein (GFP or mCherry) under the *sra-6* (ASH ablation) or *nlp-12* (DVA ablation) promoters. ASH or DVA ablations were confirmed after locomotion analysis by fluorescence microscopy.

### Aldicarb assay

Sensitivity to aldicarb was determined by analyzing the time course of paralysis following treatment with 1 mM aldicarb (Sigma-Aldrich) as previously described [42]. Briefly, movement of animals was assessed by prodding animals with a platinum wire every 10 minute following exposure to aldicarb. 20–30 animals were tested for each trial. For the capsaicin treatment (Fig 4J), adult animals were transferred to NGM plates containing 50 μM capsaicin (with food), treated with capsaicin for 2–3 hours, and assayed for their paralysis on 1 mM aldicarb plates containing 50 μM capsaicin.

### Electrophysiology

Electrophysiology was performed on dissected adult worms as previously described [43]. Worms were superfused in an extracellular solution containing 127 mM NaCl, 5 mM KCl, 26 mM NaHCO_3_, 1.25 mM NaH_2_PO_4_, 20 mM glucose, 1 mM CaCl_2_, and 4 mM MgCl_2_, bubbled with 5% CO_2_, 95% O_2_ at 20°C. Whole cell recordings were carried out at –60 mV using an internal solution containing 105 mM CsCH_3_SO_3_, 10 mM CsCl, 15 mM CsF, 4mM MgCl_2_, 5mM EGTA, 0.25mM CaCl_2_, 10mM HEPES, and 4 mM Na_2_ATP, adjusted to pH 7.2 using CsOH. Under these conditions, we only observed endogenous acetylcholine EPSCs. To record GABAergic postsynaptic currents, the holding potential was 0 mV, at which we only observe mIPSCs. All recording conditions were as described [44]. To record evoked EPSCs, a 0.4 ms, 30 μA square pulse was applied to a motor neuron cell body with a stimulating electrode placed near the ventral nerve cord (one muscle distance from the recording pipette). Statistical significance was determined using one-way ANOVA with Tukey test for multiple comparisons and two-tailed Student’s t test for pairwise comparison.

### Fluorescence microscopy and image analysis

Quantitative imaging of coelomocyte fluorescence was performed as previously described [11] using a Zeiss Axioskop equipped with an Olympus PlanAPO 100x (NA = 1.4) objective and a CoolSNAP HQ CCD camera (Photometrics). Worms were immobilized with 30 mg/ml BDM (Sigma). The anterior coelomocytes were imaged in L4/A lethargus (determined by absence of pharyngeal pumping), and 1 day-old adult animals. Image stacks were captured and maximum intensity projections were obtained using Metamorph 7.1 software (Universal Imaging). YFP fluorescence was normalized to the absolute mean fluorescence of 0.5 mm FluoSphere beads (Molecular Probes). Statistical significance was determined using Kolmogorov-Smirnov test.

### Calcium imaging and analysis

Using Dermabond topical skin adhesive, individual worms were glued to 2% agarose pads in extracellular saline (145 mM NaCl, 5 mM KCl, 1 mM CaCl_2_, 5 mM MgCl_2_, 20 mM D-glucose, and 10 mM HEPES buffer [pH7.2]). To image copper and glycerol responses, single animals were placed in a perfusion chamber (RC-26GLP,Warner Instruments) under a constant flow rate (0.4 ml min^-1^) of buffer using a perfusion pencil (AutoMate). Outflow was regulated using a peristaltic pump (Econo Pump, Bio-Rad). 10mM CuCl_2_ (copper(II)chloride dihydrate, Sigma) or 500mM glycerol (Fisher) were delivered using the perfusion, pencil and switch between control and stimulus solutions was done using manually controlled valves. Solutions contained either 10mM CuCl_2_ in M13 buffer or 500mM glycerol in 40mM NaCl, 1 mM MgSO_4_, 1 mM CaCl_2_ and 5 mM KPO_4_. The stimulus was delivered for 10 seconds starting on the 10^th^ second from the beginning of the movie. Optical recordings were performed on a Zeiss Axioskop 2 upright compound microscope equipped with a Dual View beam splitter and a Uniblitz Shutter. Images were recorded at 10 Hz using an iXon EM camera (Andor Technology) and captured using IQ1.9 software (Andor Technology). For ratiometric imaging, ROI_Y_ tracked the neuron in the yellow channel, and in the cyan channel, ROI_C_ moved at a fixed offset from ROI_Y_. F was computed as F_Y_/F_C_ following a correction for bleed through. No correction for bleaching was required. Ratio changes were detected and parametrized using scripts for MATLAB (The Mathworks). Briefly, the scripts average the F value for 5 preceding and including the marked start stimulus frame (F_0_) and the 5 frames centered on the marked peak frame (F_1_). ΔF was equal to (F_1_—F_0_) / F_0_ x 100. Touch-evoked calcium responses in PLM neurons were analyzed as previously described [11]. Statistical significance was determined using one-way ANOVA with Tukey test for multiple comparisons.

## Supporting Information

S1 Fig Stimulus-evoked EPSCs and endogenous IPSCs are normal in *npr-1* adults. Stimulus-evoked EPSCs (A-B) and mIPSCs (C-E) were recorded from body wall muscles of adult worms for the indicated genotypes. Averaged traces of stimulus-evoked EPSCs (A), representative traces of mIPSCs (C), and summary data are shown (B, D, and E). The number of animals analyzed is indicated for each genotype. Error bars indicate SEM (ns, not significant).
(TIF)

S2 Fig Time course of paralysis of animals treated with 1 mM aldicarb. Time courses (120 min) of paralysis of worms on 1 mM aldicarb were plotted for the indicated genotypes. The number of trials is indicated in the parentheses for each genotype. (A) The *npr-1* aldicarb hypersensitivity was rescued by transgenes expressing NPR-1 in the RMG circuit (RMG rescue, *flp-21* promoter) but not by those expressed in GABAergic neurons (GABA rescue, *unc-25* and *unc-30* promoters). (B-C) The *npr-1* aldicarb hypersensitivity was blocked by mutations inactivating TAX-4/CNG channels or OCR-2/TRPV channels. (D-E) *npr-1* aldicarb hypersensitivity was not abolished by mutations inactivating PDF-1 or PDFR-1, (F) but was suppressed by mutations inactivating EAT-4/VGLUT. (G) Capsaicin treatment (2–3 hours) increased aldicarb sensitivity in transgenic animals expressing TRPV1 in ASH neurons, but not in wild type controls.
(TIF)

S3 Fig Transgenic expression of EAT-4 or GLR-2 in WT worms has no effect on lethargus quiescence. Locomotion behavior of single worms during the L4/A lethargus (A-B) was analyzed in the indicated genotypes. Average motile fraction (A), and average locomotion velocity (B) are plotted. Transgenes that re-instated lethargus quiescence defects in *eat-4;npr-1* (*sra-6* or *mec-4* promoted EAT-4, Fig 3) or *glr-2;npr-1* (*gcy-28d* or *nlp-12* promoted GLR-2, Fig 5) double mutants had no effect on lethargus quiescence in wild type worms. The number of animals analyzed is indicated for each genotype.
(TIF)

S4 Fig NPR-1 is required for the decreased glycerol-evoked calcium transients in ASH during L4/A lethargus. Glycerol-evoked calcium transients in ASH were analyzed in L4, L4/A, and adults of the indicated genotypes using cameleon as a calcium indicator. Averaged responses (A, C), and the amplitudes of individual trials (B, D) are shown for each genotype. Each trace represents the average percentage change in YFP/CFP fluorescence ratio. The light tan rectangle indicates the duration for which 500 mM glycerol was applied. Dark gray shading of each trace indicates SEM of the mean response. (A-B) Glycerol-evoked calcium transients in ASH neurons were significantly reduced during L4/A lethargus, and this effect was abolished in *npr-1* mutants. (C-D) This defect during L4/A lethargus was rescued by transgenes expressing NPR-1 in the RMG circuit (RMG rescue, *flp-21* promoter) or in ASH neurons (ASH rescue, *sra-6* promoter). Values that differ significantly are indicated (***, *p* <0.001; ns, not significant).
(TIF)

S5 Fig GLR-1 and NMR-1 glutamate receptors are not required for the increased locomotion in *npr-1* adults. Locomotion behavior of single adult worms was analyzed in the indicated genotypes. Average locomotion velocity (A) is plotted. (A) The locomotion defect in *npr-1* adults was not suppressed by mutations inactivating *glr-1* or *nmr-1* glutamate receptors. The number of animals analyzed is indicated for each genotype. Error bars indicate SEM. Values that differ significantly are indicated (ns, not significant).
(TIF)

S6 Fig PLM touch sensitivity is increased in *npr-1* mutants. Touch-evoked calcium transients in PLM were analyzed using cameleon as a calcium indicator. Responses were analyzed in adult animals. Averaged responses (A) and the amplitudes of individual trials (B) are shown for each genotype. Each red trace represents the average percentage change in YFP/CFP fluorescence ratio. The black triangle indicates the time at which the mechanical stimulus was applied. Gray shading indicates the response SEM. Touch-evoked calcium transients in adult PLM neurons were significantly larger in *npr-1* mutants.
(TIF)
