# Supplementary figures and images for: Sensory Neurons Arouse C. elegans Locomotion via Both Glutamate and Neuropeptide Release
Source: PLoS Genet. 2015 Jul 8;11(7):e1005359. doi: 10.1371/journal.pgen.1005359 (PMC4495980; doi:10.1371/journal.pgen.1005359)

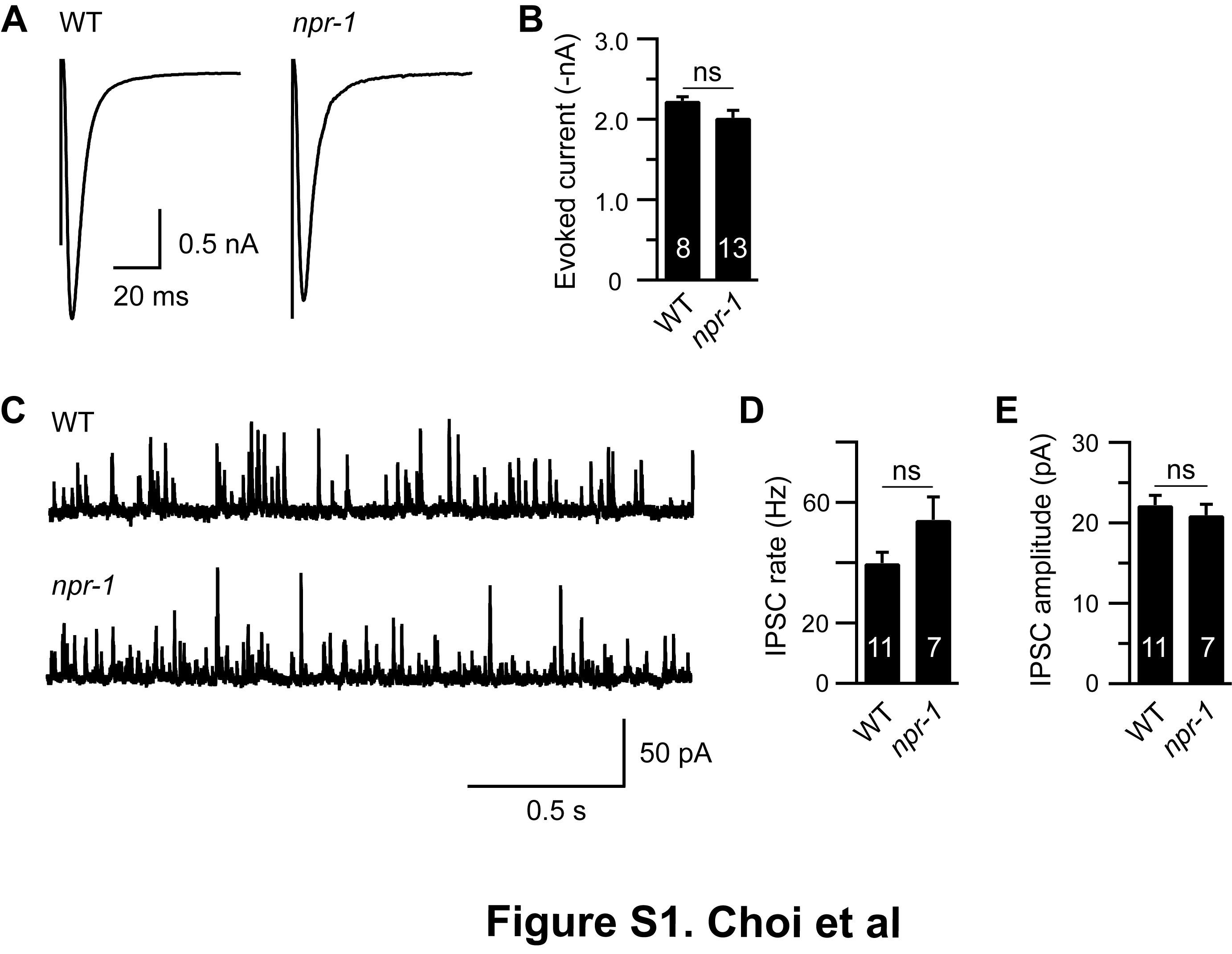

Supplement: S1 Fig — Stimulus-evoked EPSCs (A-B) and mIPSCs (C-E) were recorded from body wall muscles of adult worms for the indicated genotypes. Averaged traces of stimulus-evoked EPSCs (A), representative traces of mIPSCs (C), and summary data are shown (B, D, and E). The number of animals analyzed is indicated for each genotype. Error bars indicate SEM (ns, not significant). (TIF) [file pgen.1005359.s001.tif]

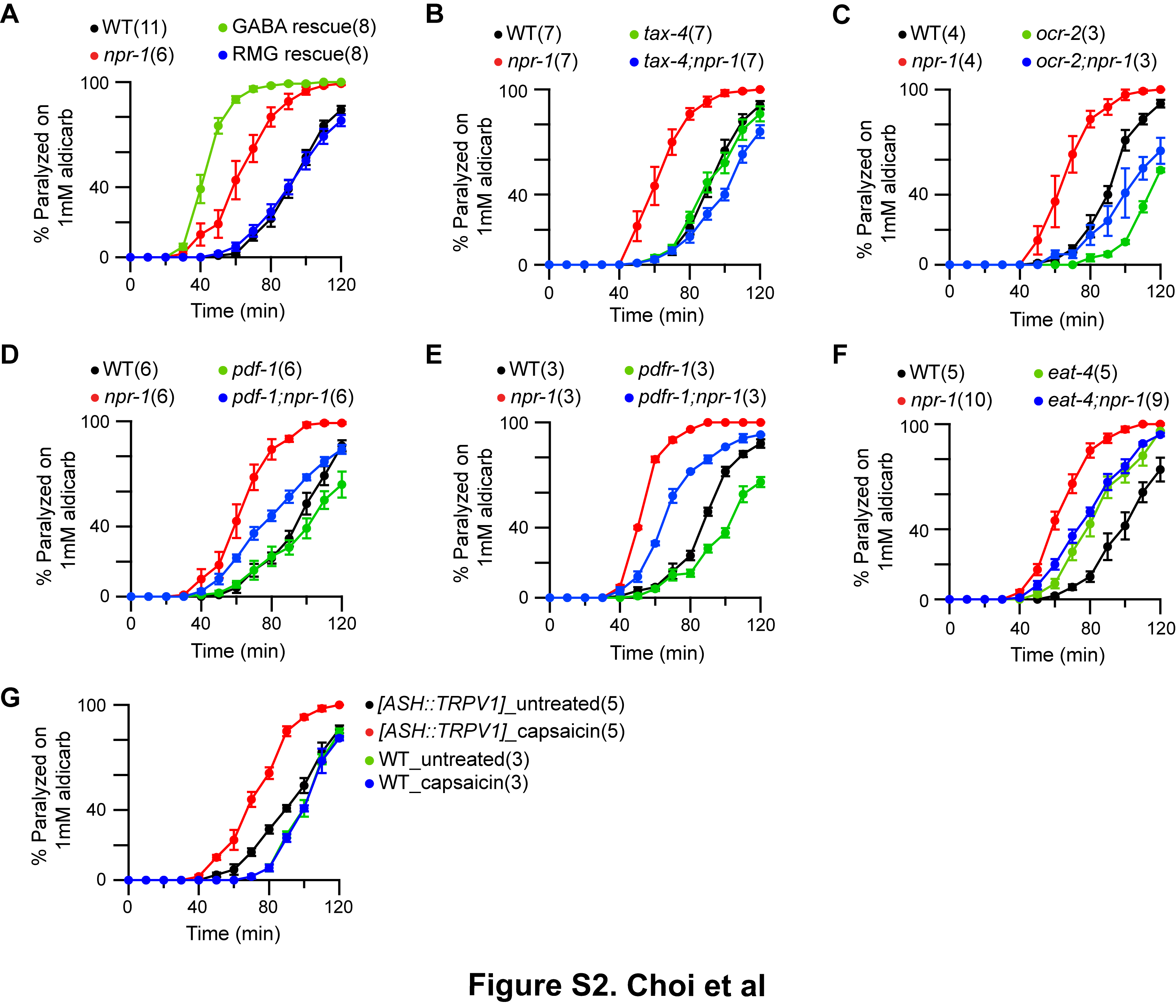

Supplement: S2 Fig — Time courses (120 min) of paralysis of worms on 1 mM aldicarb were plotted for the indicated genotypes. The number of trials is indicated in the parentheses for each genotype. (A) The npr-1 aldicarb hypersensitivity was rescued by transgenes expressing NPR-1 in the RMG circuit (RMG rescue, flp-21 promoter) but not by those expressed in GABAergic neurons (GABA rescue, unc-25 and unc-30 promoters). (B-C) The npr-1 aldicarb hypersensitivity was blocked by mutations inactivating TAX-4/CNG channels or OCR-2/TRPV channels. (D-E) npr-1 aldicarb hypersensitivity was not abolished by mutations inactivating PDF-1 or PDFR-1, (F) but was suppressed by mutations inactivating EAT-4/VGLUT. (G) Capsaicin treatment (2–3 hours) increased aldicarb sensitivity in transgenic animals expressing TRPV1 in ASH neurons, but not in wild type controls. (TIF) [file pgen.1005359.s002.tif]

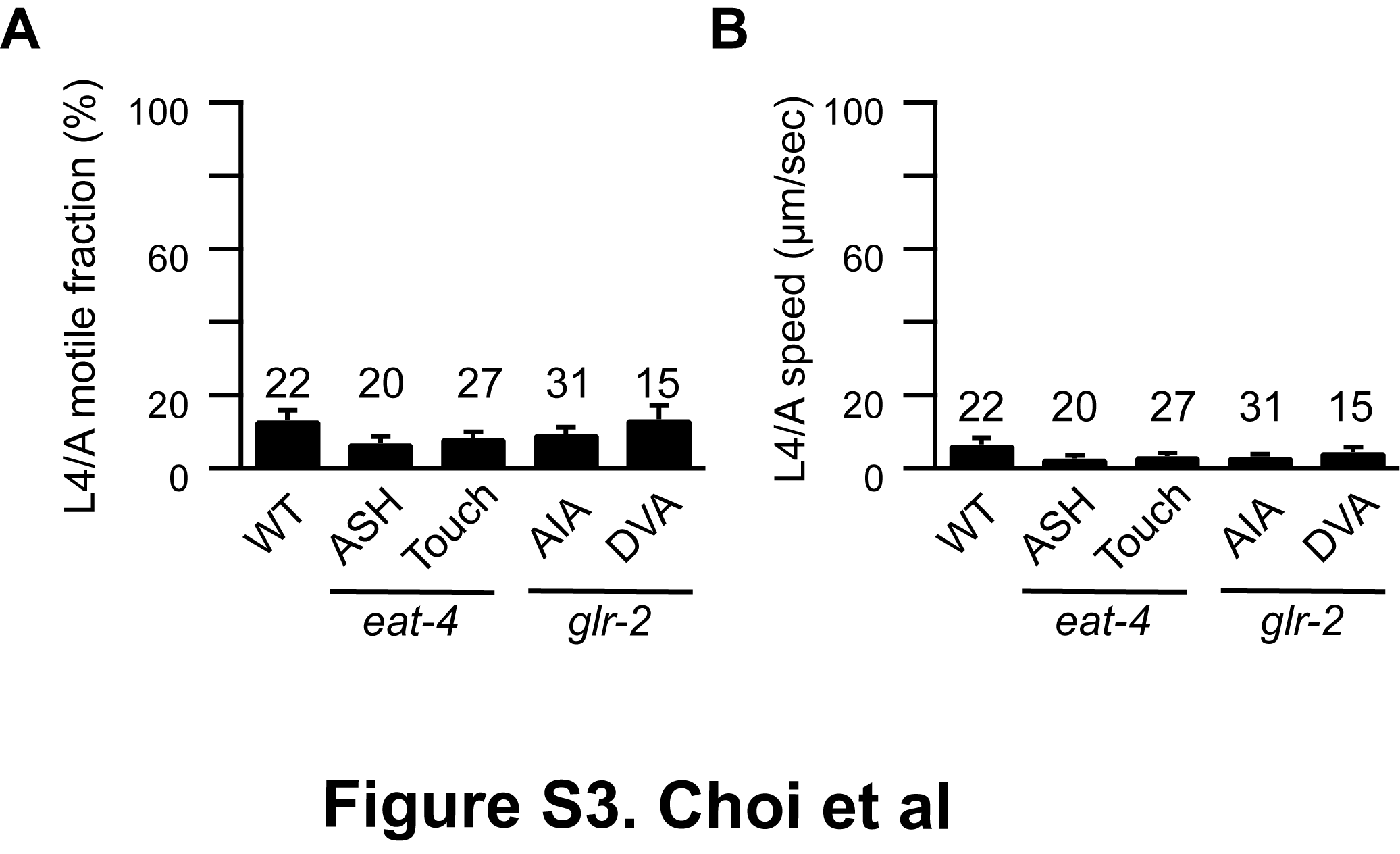

Supplement: S3 Fig — Locomotion behavior of single worms during the L4/A lethargus (A-B) was analyzed in the indicated genotypes. Average motile fraction (A), and average locomotion velocity (B) are plotted. Transgenes that re-instated lethargus quiescence defects in eat-4;npr-1 (sra-6 or mec-4 promoted EAT-4, Fig 3) or glr-2;npr-1 (gcy-28d or nlp-12 promoted GLR-2, Fig 5) double mutants had no effect on lethargus quiescence in wild type worms. The number of animals analyzed is indicated for each genotype. (TIF) [file pgen.1005359.s003.tif]

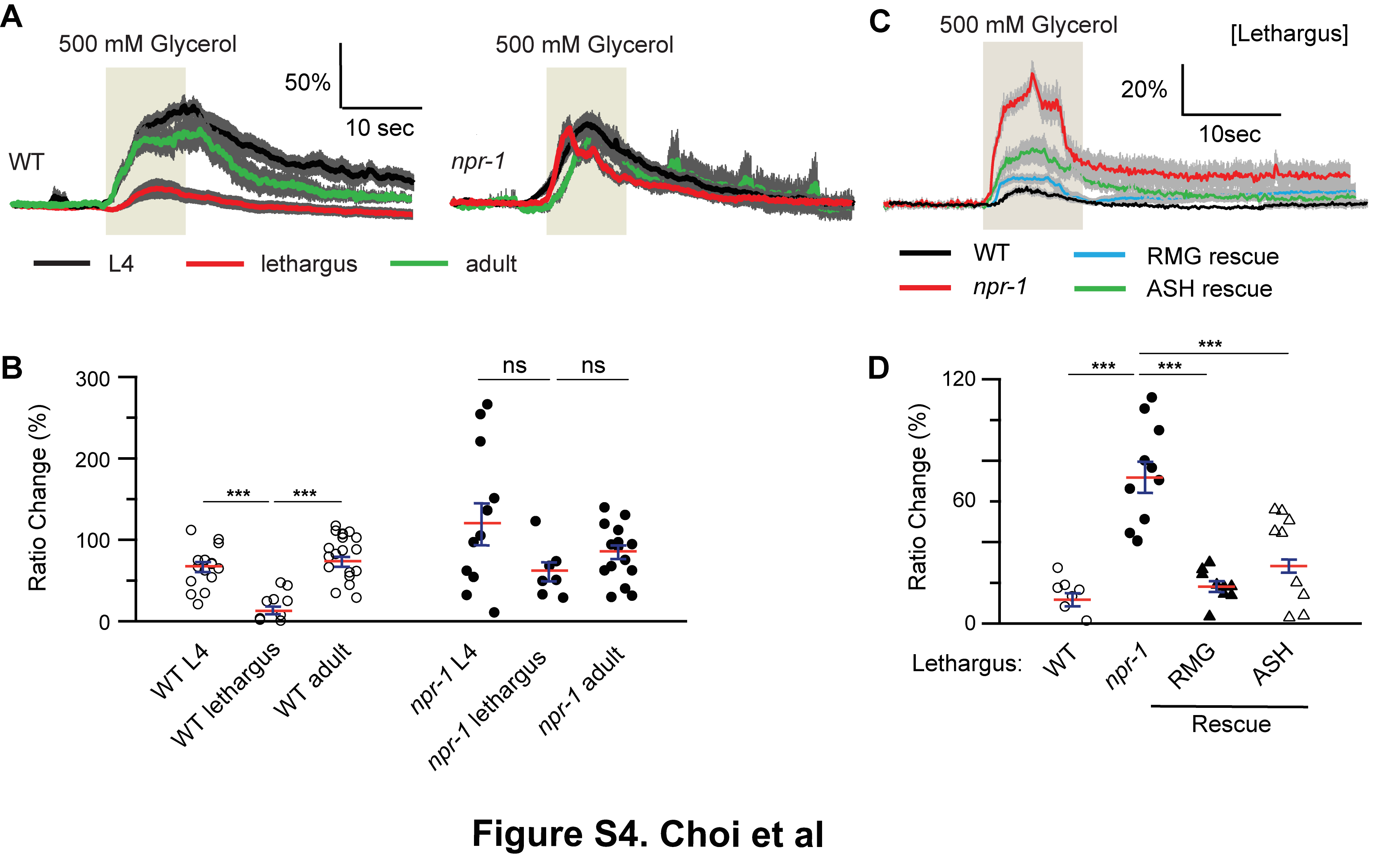

Supplement: S4 Fig — Glycerol-evoked calcium transients in ASH were analyzed in L4, L4/A, and adults of the indicated genotypes using cameleon as a calcium indicator. Averaged responses (A, C), and the amplitudes of individual trials (B, D) are shown for each genotype. Each trace represents the average percentage change in YFP/CFP fluorescence ratio. The light tan rectangle indicates the duration for which 500 mM glycerol was applied. Dark gray shading of each trace indicates SEM of the mean response. (A-B) Glycerol-evoked calcium transients in ASH neurons were significantly reduced during L4/A lethargus, and this effect was abolished in npr-1 mutants. (C-D) This defect during L4/A lethargus was rescued by transgenes expressing NPR-1 in the RMG circuit (RMG rescue, flp-21 promoter) or in ASH neurons (ASH rescue, sra-6 promoter). Values that differ significantly are indicated (***, p <0.001; ns, not significant). (TIF) [file pgen.1005359.s004.tif]

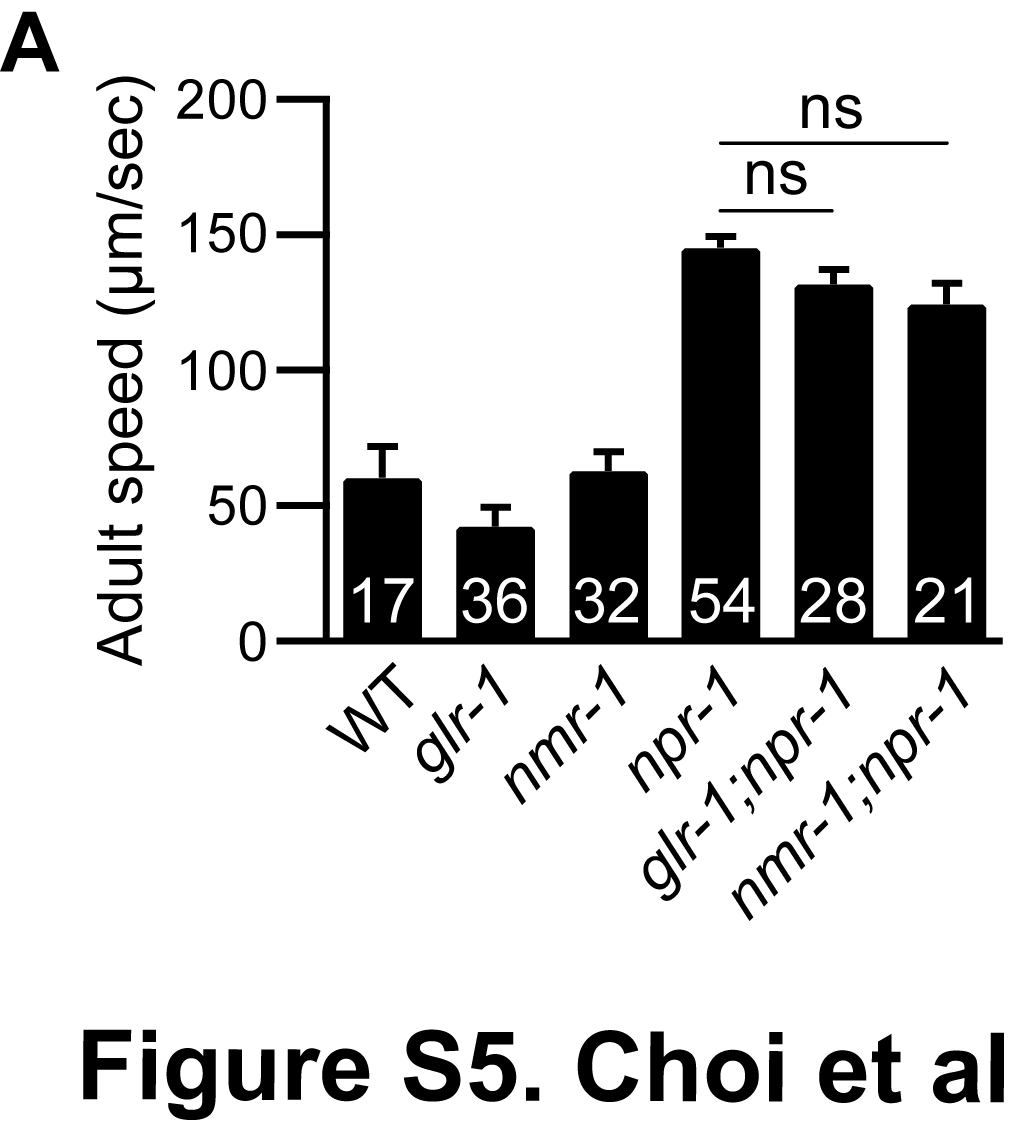

Supplement: S5 Fig — Locomotion behavior of single adult worms was analyzed in the indicated genotypes. Average locomotion velocity (A) is plotted. (A) The locomotion defect in npr-1 adults was not suppressed by mutations inactivating glr-1 or nmr-1 glutamate receptors. The number of animals analyzed is indicated for each genotype. Error bars indicate SEM. Values that differ significantly are indicated (ns, not significant). (TIF) [file pgen.1005359.s005.tif]

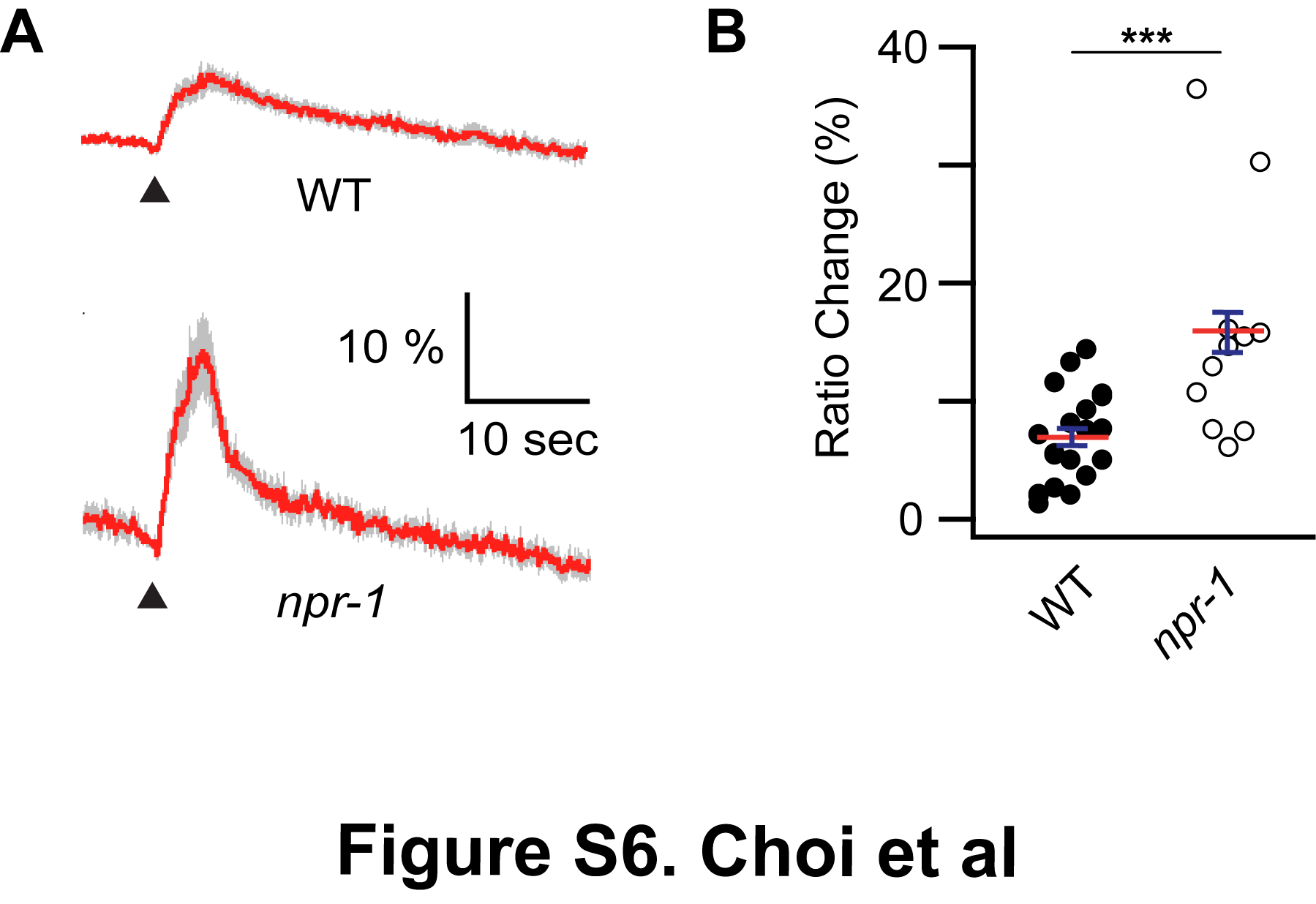

Supplement: S6 Fig — Touch-evoked calcium transients in PLM were analyzed using cameleon as a calcium indicator. Responses were analyzed in adult animals. Averaged responses (A) and the amplitudes of individual trials (B) are shown for each genotype. Each red trace represents the average percentage change in YFP/CFP fluorescence ratio. The black triangle indicates the time at which the mechanical stimulus was applied. Gray shading indicates the response SEM. Touch-evoked calcium transients in adult PLM neurons were significantly larger in npr-1 mutants. (TIF) [file pgen.1005359.s006.tif]
